# Deciphering the Human Germinal Center: A Review of Models to Study T–B Cell Interactions

**DOI:** 10.1002/eji.202451460

**Published:** 2025-02-11

**Authors:** Elisa Fleischmann, Vera Middelkamp, Theo van den Broek

**Affiliations:** ^1^ Center for Translational Immunology University Medical Center Utrecht Utrecht University Utrecht The Netherlands

## Abstract

Interactions between T‐ and B cells in the germinal center reaction are instrumental for the initiation, maintenance, and downregulation of the human adaptive immune response, leading to the production of antigen‐specific antibodies and long‐lasting immunological memory. Replicating the human immune system remains challenging, with an over‐reliance on animal models with limited translational accuracy. There is an increasing need for new tools that accurately model human immune function. This review evaluates existing 2D and 3D in vitro and ex vivo human models for their ability to reproduce the germinal center reaction, with a particular focus on T‐ and B‐cell interaction. We conclude that although current models are able to replicate certain features of the germinal center reaction, no current model is able to completely replicate the complex human GC process. We outline the challenges in recreating a fully functional germinal center and suggest future directions of research to improve existing models, ultimately bringing us closer to completely reproducing the human lymph node.

## Graphical abstract

This review examines 2D and 3D human models for replicating germinal center reactions, focusing on T–B cell interactions. While current models capture some features, none fully replicate the complexity of the human germinal center. We highlight challenges and propose future directions to enhance model accuracy and better mimic human immune responses.

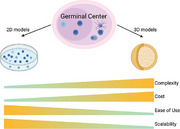

## 1 INTRODUCTION

The generation of an effective humoral immune response and establishment of enduring immunological memory relies on the production of high‐affinity antibodies [1]. High‐affinity antibody generation is dependent on a specific process in lymphoid structures, the germinal center process (GC). The GC is a complex system involving many different cell types and interactions, including the prominent T‐ and B‐cell interaction, that is crucial for the initiation, maintenance, and regulation of the GC [2]. Understanding the quality and quantity of T‐ and B‐cell interactions is essential, as these factors directly influence the outcome of the GC process.

**FIGURE 1:**
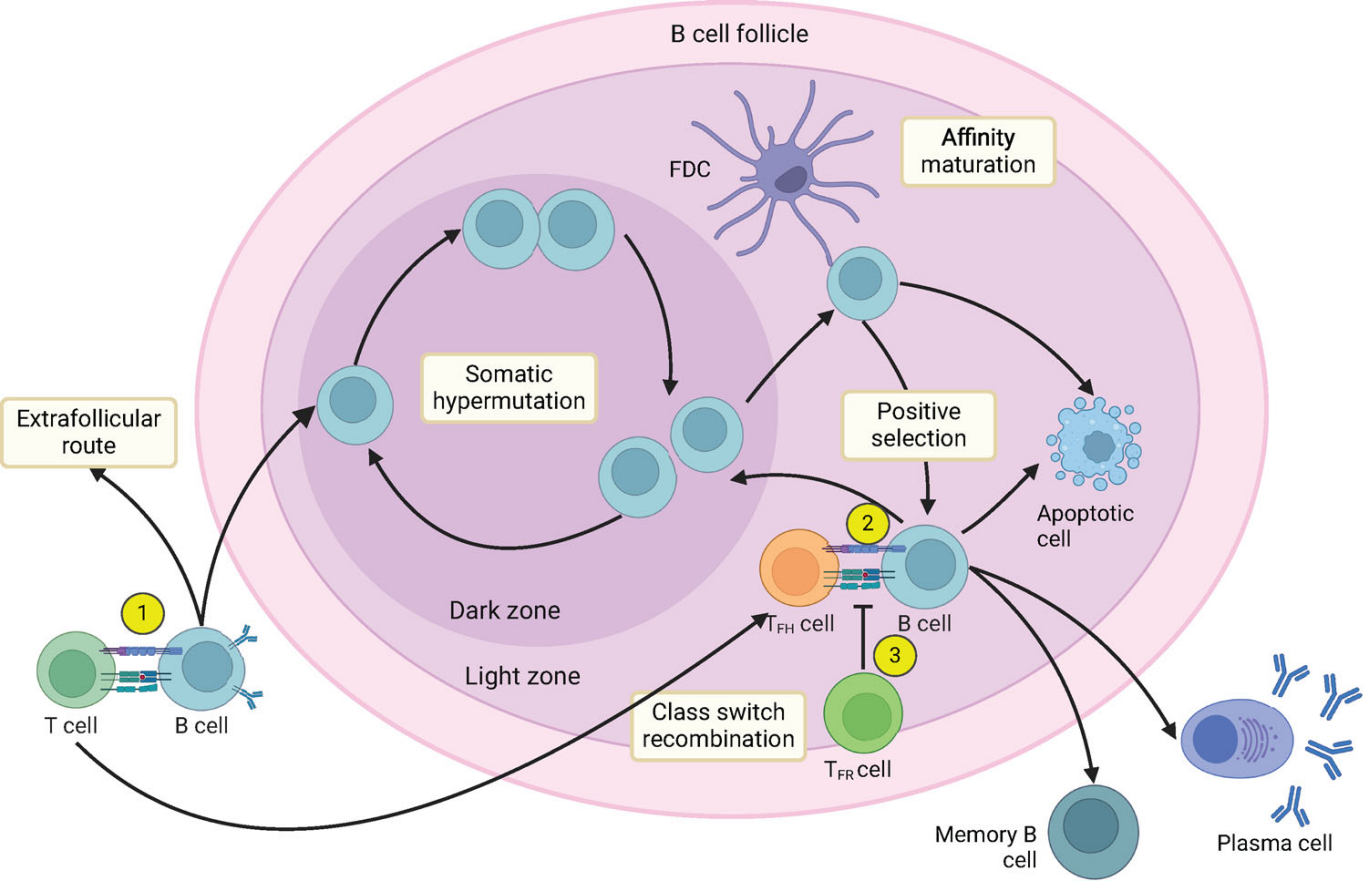
The germinal center (GC) reaction. Simplified schematic representation of the germinal center (GC) process and important T:B cell interactions [4, 9]. (1) At the T:B cell border of the lymph node, activated B cells interact with T helper cells, which provide them with survival and costimulatory signals to either initiate the GC response or undergo the extrafollicular route. (2) Positively selected B cells interact with T follicular helper (T_FH_) cells, which results in B cells in cyclic re‐entry, terminal differentiation, or apoptosis. (3) Regulatory follicular T cells (T_FR_) are involved in the regulation of the GC process.

GCs are microanatomical structures within B cell follicles of secondary lymphoid tissues that form in response to B cell exposure to an antigen [3]. B cells can directly bind to soluble antigens or to antigens presented by antigen‐presenting cells, and upon activation, B cells migrate to the border of the follicle, called the T:B border [1, 2, 4]. Here T cells provide B cells with survival and costimulatory signals resulting in B cells that initiate the GC response or an extrafollicular response (Figure 1) [1, 2].

A mature GC is divided into two sites based on cell density: the light zone (LZ) and the dark zone (DZ). In the DZ, B cells proliferate and undergo de novo somatic hypermutation (SHM): insertion of random mutations into the immunoglobulin variable genes, altering the affinity and stability of the antigen‐binding site of the B cell [4, 5, 6, 7, 8]. Subsequently, these mutated GC B cells migrate to the LZ, where the antigen‐binding affinities of the B cells are tested through interactions with follicular dendritic cells (FDCs) [4, 5, 6, 8, 9]. FDCs capture and present antigens to the B cells. Only B cell clones with relatively high affinity to the presented antigen collect it, enabling these B cells to interact with T follicular helper (T_FH_) cells and receive survival signals in a process called positive selection [1, 2, 4, 5]. Lower affinity B cells are unable to collect the antigen, acquire insufficient survival signals, and undergo apoptosis [4, 5, 9, 10]. The selected high‐affinity B cells are then either stimulated toward cyclic re‐entry, where the B cells re‐enter the DZ for further proliferation and SHM, or toward terminal differentiation, where they develop into mature high‐affinity antibody‐secreting plasma cells (PCs) and memory B cells [1, 4, 5, 6, 8, 11]. Regulation of the GC process is a complex and multicellular process that involves regulatory follicular T cells (T_FR_), that can influence the T_FH_:GC B cell interaction and subsequent output [4, 5, 6, 12].

In both mice and humans, GC B cells are characterized by an IgD‐, CD95+ (Fas), and PNA+ cell surface phenotype. However, murine GC B cells additionally express GL7 and lack CD38, whereas human GC B cells are CD10+ and CD38+. Both murine and human GC B cells share expression of the transcription factor BCL6 and chemokine receptor CXCR5, with CXCR4+ cells localized in the dark zone, where somatic hypermutation occurs, and CD86+ cells in the light zone of the germinal center [13, 14, 15]. Activation‐induced cytidine deaminase (AID) is commonly used as a marker for class‐switch recombination (CSR), SHM, and as an identifier of GC B cells. However, CSR can occur outside of the follicles before GC formation and prior to AID expression 16]. Activated B cells already express AID, albeit at lower levels compared with GC B cells, where it is more prominently involved in SHM [13, 16]. Therefore, AID expression is not exclusively associated with GC B cells or active CSR.

While murine models have led to important insights into the GC process and T:B cell interactions, their translation to humans is impaired [3, 10, 17, 18]. Therefore, human models are indispensable in accurately assessing these interactions, providing insights that are crucial for advancing our knowledge of humoral immune responses and improving therapeutic strategies. The development of in vitro and ex vivo models, such as immune organoids or 3D culture methods, could overcome these limitations, and more accurately model human immune responses [3]. However, this is complicated by the fact that GC B cells, unlike other B cell subtypes, undergo apoptosis in culture after a few hours, without the addition of CD40 [19]. In this review, we compare and evaluate the current systems and models to guide their specific applications to further advance our understanding of the human GC reaction and mechanisms of protective immunity.

## 2 2D MODELS

**FIGURE 2:**
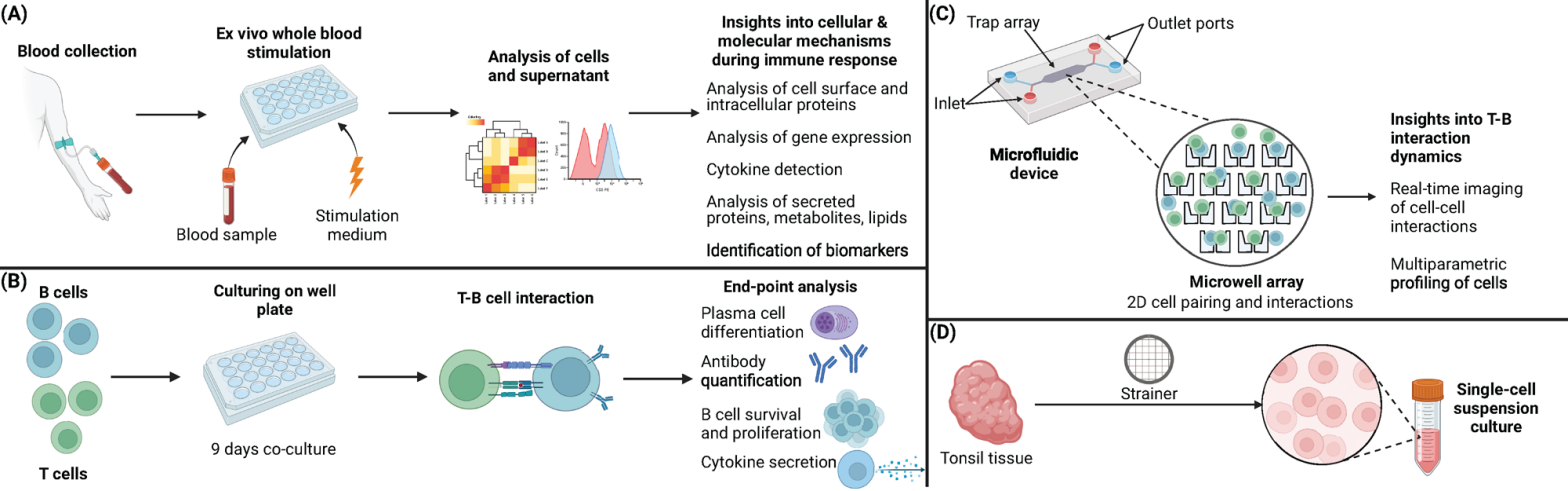
Overview of 2D models. (A) Procedure for whole‐blood assays. (B) T–B cell 2D coculture procedure. (C) Setup of the microfluidic device, including inlet and outlet ports and the trap array. (D) Ex vivo tissue cultures from single‐cell suspensions.

Two‐dimensional (2D) assays have been the go‐to method to culture cells since the early 1900s, and are associated with simplicity, speed, practicality, and cost‐effectiveness [17, 20]. In vitro and ex vivo models may provide a reliable, reproducible, and ethical alternative to animal models and recent advancements have enabled the development of 2D models for analyzing controlled cell‐cell interactions, including those involving T and B lymphocytes [17, 21]. These methods include whole‐blood assays, ex vivo tissue cultures, isolated cell cocultures, and microfluidic pairing devices (Figure 2, Table 1). Whole blood assays use fresh anticoagulated human blood, preserving the blood's physiologic environment and allowing interaction among all circulating cell types, but making it unable to directly study GC B and T_FH_ cells [17, 22, 23]. For 2D ex vivo tissue cultures, cells are extracted from the tissue into single‐cell suspensions and cultured in well plates [24]. Similarly, in isolated cell cocultures, isolated T‐ and B cells are cultured together, for example in the presence of superantigen‐like Staphylococcal enterotoxin B to evaluate the potential of CD4+ T cell (incl T_FH_) to help with B cell survival, proliferation, differentiation and antibody production [24, 25, 26]. Lastly, in microfluidic pairing, hundreds of T and B cells are trapped and paired in a controlled microenvironment on high throughput chips, to perform real‐time imaging of the interactions and multiparametric profiling of the cells [27, 28, 29, 30]. However, as the microenvironments in these models lack ECM and other cell types that support T–B interactions and promote the survival of these immune cells, they cannot fully recreate the in vivo lymph node [24]. Therefore, these models are limited to short‐term studies lasting only a few days. Consequently, they are unlikely to support de novo GC reactions, such as SHM or CSR, which typically require about a week to occur. This limitation reduces the translational accuracy of these models [10, 24].

**TABLE 1:** Summary and comparison of discussed 2D models for studying T–B interactions and the features that are captured with it.

	Origin T & B cells	Diverse cell types	Antibody production^a^	Cytokines and other soluble mediators	Plasma cell formation	Regulated fluid flow	GC formation	Sources
Whole‐blood assays	Whole blood	✓	✓	✓	X	X	X	[18, 23, 24]
Ex vivo tissue cell culture	LN	✓	X	✓	X	X	X	[25]
2D cocultures	PBMCs/LN	X (only if added)	✓	✓	✓	X	X	[25, 26, 27]
Microfluidic devices	PBMCs	X (only if added)	X	✓	X	✓	X	[28, 29, 30, 31]

*Note*: ✓ indicates present and X indicates absent or unknown in current literature.

Abbreviations: LN, lymph node; PBMCs, peripheral blood mononuclear cells.

^a^Antigen‐specific antibody production following immunization or exposure to an antigen.

## 3 Models with Both 2D and 3D Aspects

### 3.1 Modular Immune in Vitro Construct

**FIGURE 3:**
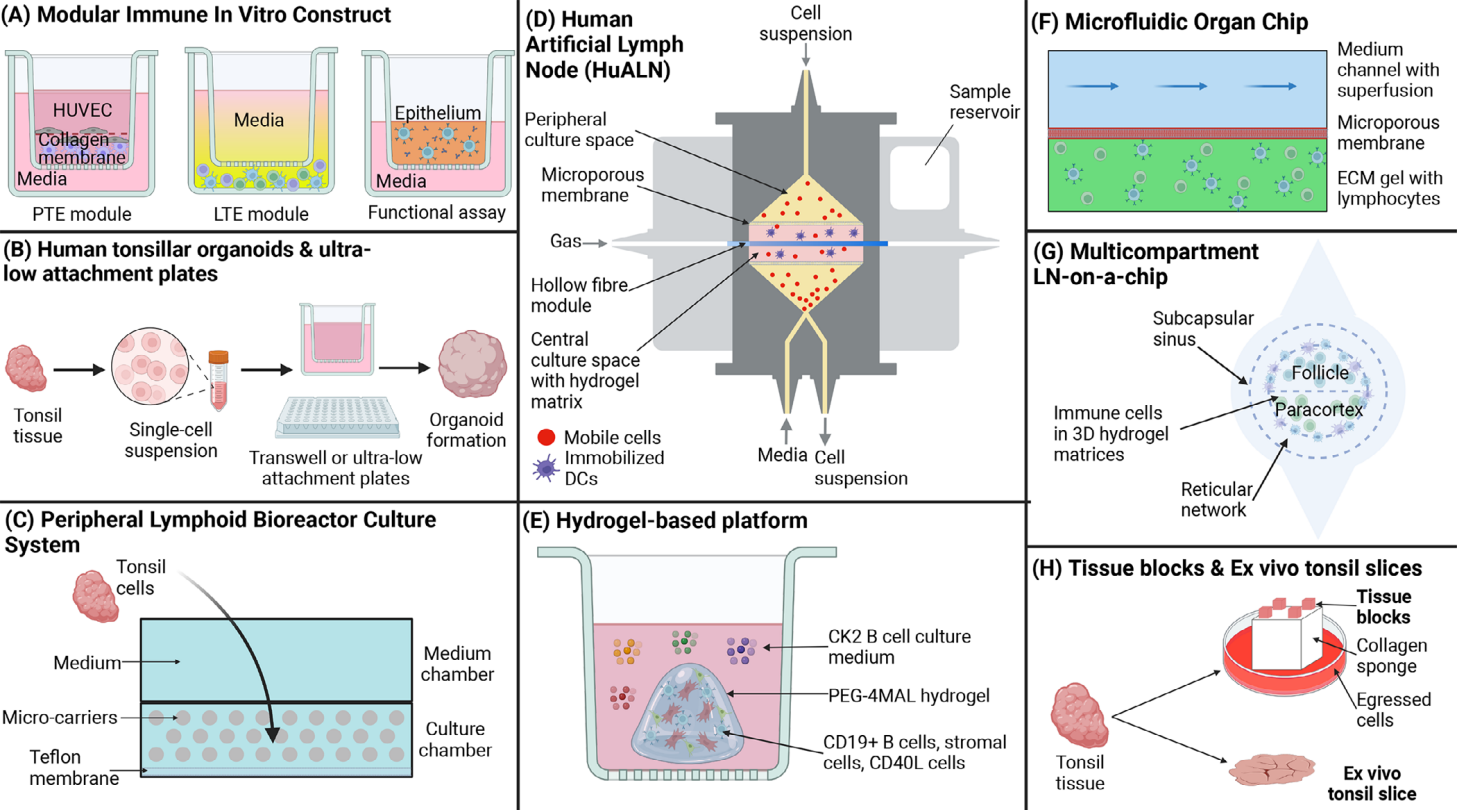
Overview of 3D models. (A) MIMIC system, (B–E) overview of organoids, (F, G) organ‐on‐a‐chip systems. (H) Ex vivo lymphoid tissue culture systems.

The modular immune in vitro construct (MIMIC) is a human cell‐based platform using sera and PBMCs to generate a co‐culture in transwell plates and consists of three modules (Figure 3A) [31, 32, 33]. A 3D peripheral tissue‐equivalent (PTE) module mimics the skin and innate immune responses. Stimulated PBMCs are seeded onto an endothelial cell layer that acts as a membrane, and APCs are harvested from the other side of the membrane [33, 34, 35]. A 2D lymphoid tissue equivalent (LTE) module replicates the LN adaptive immune responses, especially spatiotemporal kinetics like T–B cell interactions. Here, the APCs from the LTE are co‐cultured with T and B cells isolated directly from PBMCs [33, 34, 35]. Third, the functional assay module then assesses the output of the interactions between immune cells and vaccine components [35]. The system allows for various study configurations and high‐throughput testing [31, 36].

The MIMIC system evaluates immune responses to vaccines, assessing both innate and adaptive immunity, including recall responses [17, 31, 32, 33, 35, 36, 37]. It can investigate early immune events and has shown age‐associated changes in immune responses after influenza vaccination, reflecting its ability to recreate patient‐specific, in vivo immune responses [31, 35, 37]. T–B cell interaction is evident from the production of antigen‐specific antibodies from B cells in response to detected T‐cell activation [31]. However, IgG antibodies were produced from memory B cells and not de novo [31, 32, 38]. Notably, as the PTE module can be viewed as a 3D, the LTE module is a 2D coculture and the MIMIC platform does not seem to form any T‐ or B‐cell clusters that could indicate GC formation, so we here characterize it in between 2D and 3D models.

## 4 3D MODELS

In theory, three‐dimensional (3D) models better mimic the tissue structure in vivo than 2D models and permit the regulated incorporation of the spatiotemporal dynamics and biophysical cues that are essential for the survival, growth, and functions of cells within the LN [21, 34, 39, 40, 41]. This includes biochemical gradients and biomechanical forces, as well as fluid flow, spatial organization, extracellular matrix (ECM) components, and stromal populations [17, 21, 34, 36, 40]. For example, LN stromal cells are found to be essential for lymphocyte survival, migration, and maintaining LN structure and function [42, 43]. Moreover, ECM proteins are critical in GC B cell reactions and antigen‐specific antibody production, and many signaling molecules require binding to the ECM to function, so 3D culture models are essential to study GC functionality [3]. However, current 3D complex in vitro models have low throughput rates, higher costs, lack validation and compatibility with standard assessment techniques, and require specialized tissue engineering training and equipment, which limits their commercial use [17, 36]. 3D models encompass a variety of systems, ranging from in vitro models like organoids and organ‐on‐a‐chip devices with microfluidic and microfabrication techniques, to ex vivo models of whole human LN tissue slices, each with distinct characteristics that will be discussed (Table 2).

**TABLE 2:** Summary of existing 3D models that attempt to replicate the germinal center reaction and the features that are captured with it.

	Origin T & B cells	Regulated fluid flow	Germinal center formation	Class switch recombination	Somatic hypermutation	Antibody production^a^	Response to neo‐antigen	Sources
Organoids								
HuALN	PBMC	✓	X, T and B cell clusters detected	X	X	✓	X	[65, 66, 67, 68, 69]
Peripheral lymphoid bioreactor system	LN	✓	X, Clusters with B‐cell enriched areas	✓	X	✓	✓	[62]
MIMIC	PBMC	X	X	X	X	✓	✓	[32, 33, 34]
Human tonsillar organoid	LN	X	✓, Evidence of LZ/DZ clusters	✓	✓	✓	✓	[19, 60, 73]
Ultra‐low attachment plates	LN	X	✓, Evidence of LZ/DZ clusters	✓	✓	✓	X	[47]
Hydrogel‐ based organoid platform	PBMC	X	X, B cell clusters formed	✓	X	X	X	[3]
Organ‐on‐a‐chip								
Microfluidic LN‐on‐a‐chip	PBMC	✓	X, Evidence of follicle formation	✓	X, AID expression found	✓	X	[64]
Multicompartment LN‐on‐a‐chip	HSCs	✓	X, LN compartments formed	X	X	X	X	[70, 71]
Lymphoid organ chip	PBMC	✓	X, Cluster formation without LZ/DZ separation	✓	X, AID expression found	✓	X	[72]
Ex vivo cultures								
Tissue blocks	LN	X	✓	X	X	✓	X	[74, 75, 76, 77, 78]

*Note*: ✓ indicates present and X indicates absent or unknown in current literature. Abbreviations: LN, lymph node; PBMC, peripheral blood mononuclear cells; HSC, hematopoietic stem cells; LZ, light zone; DZ, dark zone.

^a^Antigen‐specific antibody production following immunization or exposure to an antigen.

### 4.1 Organoids

Organoids are in vitro miniaturized versions of human tissue or organs that assemble into 3D and mimic key structural and functional features of the tissue or organ [17, 41, 44]. Organoid cultures using biomaterials allow accurate control over parameters, including its microenvironment and cell and matrix makeup, and still allow high throughput [34, 45]. Cultures can be produced at any stage of the response, enabling mechanistic experiments and the systematic assessment of cellular changes in these human tissues over time, multiple conditions can be tested within the same donor, and effects of interindividual variability can be minimized [45, 46]. Immune organoids mimicking LN functions and generating GC‐like structures have begun to be successful in murine models and have produced fundamental insights into pathways involved in GC formation and the role of T–B cell interaction [10, 47, 48, 49, 50, 51, 52, 53, 54, 55, 56, 57, 58]. Current barriers to the use of organoids include their limited longevity, with immune organoids only remaining viable for about 3 weeks, which can restrict GC reaction studies [45]. Several examples are discussed below.

#### 4.1.1 Human Tonsillar Organoids

In this model, single‐cell suspensions of tonsil tissues are created as discussed for 2D ex vivo tissue cultures, but now plated onto a permeable transwell membrane, which resulted in the cluster formation of B cell‐ and T cell‐rich aggregates indicating a GC‐like structure, with evidence of DZ and LZ organization (Figure 3B) [18]. After stimulation, these organoids induced CD4+ and CD8+ T cell activation and proliferation, B cell maturation, plasmablast differentiation, and the production of influenza‐specific antibodies. AID expression was significantly elevated in pre‐GC and GC B cells in these organoid cultures [59]. Kastenschmidt et al. adapted the human tonsil organoid model by using ultra‐low attachment plates instead of transwells to prevent cell adherence to the surface, thereby promoting the formation of organoids, and recapitulating class switching better than on the transwells (Figure 3B), although the presence of PCs was not established [45, 46, 60].

#### 4.1.2 Peripheral Lymphoid Bioreactor Culture System

A bioreactor system designed for bone marrow cultures was adapted for long‐term, functional culture of human primary cells from tonsils without distinct exogenous growth factors or activators [34, 61]. Tonsil‐derived cells, with T and B lymphocytes, are introduced into the upper culture chamber and combined with porous cellulose microspheres as scaffolds [61]. The lower culture system utilizes a diffusion barrier and a 3D scaffold to provide optimal physicochemical conditions for better cell viability (Figure 3C) [61].

The model was challenged with a variety of diverse antigens, generating active responses of antigen‐specific antibodies to both recall and neo‐antigens, revealing that the cells within the system are immunologically competent [61]. However, the frequency of these in vitro immune responses fluctuated considerably depending on the antigen, indicating that immunization methods should be further optimized [61]. This culture system supported cellular self‐organization in large cell clusters with B cell‐enriched areas, which is reminiscent of GC organization in a more simplified form [61]. However, GC B cells are short‐lived in this 3D culture, which reflects their short life span in vivo and dependence on constant interactions within the GC structure [61].

#### 4.1.3 Human Artificial Lymph Node

The model of the human artificial lymph node (HuALN) was one of the earliest models to recapitulate organ‐specific level functions [38, 62]. This bioreactor system consists of two cell culture compartments separated by a double‐layered oxygenating membrane that incorporates constant culture medium perfusion, establishing well‐defined gradients (Figure 3D). Lymphocytes, antigen‐presenting mature DCs, and stromal cells are co‐cultured in a 3D collagen‐based matrix, enabling cell–cell and cell–matrix interactions. The bioreactor incorporates controlled perfusion, oxygenation, and feeding, ensuring long‐term cell cultivation of up to 4 weeks with donor‐to‐donor variation [38, 40, 63, 64, 65, 66, 67, 68]. Therefore, these tunable parameters provide control over the system, allowing better mimicking of the in vivo tissue, but also greatly increasing complexity [38]. This bioreactor‐based model has been used to test vaccination and drug responses, detect long‐term immunogenicity of monoclonal antibodies, analyze genetic profiles, and quantify cytokine and immunoglobulin secretion [35, 65, 66, 68]. In response to viral inoculation, activated T and B cells formed micro‐organoid structures, mimicking lymphatic follicles and GCs and replicating the 3D structure of the human LN [66, 68]. B cell maturation occurs in response to T–B cell interactions, with proliferation of T cells, and later, de novo PC formation with antigen‐specific antibody secretion [64, 67]. As AID was downregulated, it is uncertain if this bioreactor‐based model can recapitulate certain aspects of the GC response, such as SHM. Further, stromal cells, involved in CSR and SHM, are absent [65, 66].

#### 4.1.4 Hydrogel‐Based Platform

To enhance in vitro GC simulation using 3D culture techniques, a hydrogel‐based human 3D lymphoid model was developed (Figure 3E) [3]. This model uses PEG‐4MAL hydrogel to replicate the 3D ECM, incorporating human B cells, irradiated murine fibroblast CD40L+ cells as a substitute for human T cells, and tonsil‐derived lymphoid stromal cells expressing fibroblast reticular cell (FRC) markers [3]. The use of a hydrogel makes the model highly tunable to biophysical parameters like stiffness and porosity, and the use of a synthetic hydrogel, rather than natural, minimizes the risk of unwanted immune reactions. The 3D environment, supplemented with cytokines, significantly improved B cell survival, proliferation, and antibody production compared with 2D cultures. Over 14 days, large B‐cell clusters formed, attaching to stromal cells, are indicative of a GC‐like structure [3]. The model showed increased plasmablasts and plasma cells, B cell differentiation, and antibody production compared with 2D cultures. Flow cytometry revealed class switching of naïve IgD+ B cells to IgG and IgA B cells, and memory B cells differentiated into IgM‐, IgG‐, or IgA‐secreting cells [3]. These findings suggest the model is valuable for studying CSR and PC differentiation. Although T‐cell interactions are simulated using CD40L and IL‐4, the presence of CSR confirms functional B‐cell activation. Further optimization of the model could use human T cells, as well as follicular DCs, and a self‐supporting system could eliminate the need for cytokine supplementation [3].

### 4.2 Organ‐On‐A‐Chip

Organs‐on‐a‐chip (OOCs) are microfluidic cell culture devices that create a 3D dynamic model by combining micrometer‐scale cell culture with continuous fluid flow. These chips permit dynamic control over the ECM, cellular interactions, concentration gradients, incorporation of different cell types, and high cell density and activity, providing a regulated microenvironment that mimics in vivo conditions [34, 35, 40, 41].

#### 4.2.1 Microfluidic LN‐on‐a‐chip

In this microfluidic LN‐on‐a‐chip, two channels are separated by a microporous membrane where human T‐ and B cells from peripheral blood self‐assemble into 3D multicellular aggregates resembling ectopic lymphatic follicles (Figure 3F) [63]. One channel contains lymphocytes in a 3D ECM gel, while the parallel channel's flow (termed superfusion) promotes GC formation. T‐ and B cells spontaneously formed ectopic lymphatic follicles, expressed CXCL13, and made cell–cell contacts. B cells, of which most were naïve, expressed AID, that is expressed on activated B cells and expressed to the highest degree on GC B cells [63]. The perfused OOC produced higher AID expression than static 3D cultures, highlighting the importance of fluid flow for immune cell function. The incorporation of autologous antigen‐presenting DCs, with perfusion of IL‐4 and CD40L, enabled naïve B cells to produce antigen‐specific IgG antibodies and cytokines in response to an influenza vaccine 63]. This setup demonstrated B cell CSR and observed PC development, although no further tests were conducted to confirm SHM 63].

#### 4.2.2 Multicompartment LN‐on‐a‐chip

A multicompartment LN‐on‐a‐chip, featuring distinct compartments to mimic in vivo architecture, allows the co‐culture of various immune cell types in their respective zones (Figure 3G). This Organ Chip recreates LN architecture, ECM components, fluid flow patterns, and cell interactions, making it useful for analyzing drug effects on cell dynamics [69, 70]. Co‐cultured T‐ and B cells moved freely within this perfused OOC, and initial DC–T cell interactions were observed [70].

Recently, Jeger‐Madiot et al. [71] developed a lymphoid organ‐on‐a‐chip consisting of human PBMCs within a 3D collagen matrix. Apart from GC‐like clusters of B and T cells and plasmablast differentiation, the model was able to capture specific antibody production upon COVID‐19 mRNA vaccine boosting, as well as donor‐specific differences in response to this vaccine boosting. Clinical translation of these findings, as well as the opportunities to apply the model to other immunological stimuli, remains to be investigated.

### 4.3 Ex Vivo Tissue Cultures

Ex vivo tissue cultures better preserve the extracellular microenvironment, cell diversity, and spatial organization of tissues compared with in vitro single‐cell suspension cultures than both 2D models and organoids or OOC preparations [38, 78]. Tonsillar tissue is frequently used to study LN function and human immunity due to its high frequency of GCs [18]. However, ex vivo cultures lack blood and lymphatic flow, leading to reduced viability, which combined with lymphocyte egression limits their use in short‐term studies, and the use of tissue sections leads to variability and heterogeneity [78].

#### 4.3.1 Tissue Blocks

Tissue blocks are cultured at the liquid‐air interface on a raft (Figure 3H). These blocks maintain their original 3D structure and cytoarchitecture, including major lymphocyte subtypes, stromal cells, and FDC cell networks [73, 74]. Their size allows for well‐defined secondary follicles with GCs, enabling cell interactions that more accurately mimic in vivo processes [73, 75]. However, studies show that follicle integrity lasts only for 3–4 days, hindering the study of de novo GC responses that typically require 1–2 weeks [73, 74, 75, 79].

#### 4.3.2 Lymph Node Tissue Slices

Tissue slice platforms allow better diffusion of oxygen and nutrients, increasing viability and longevity compared with tissue blocks, while maintaining the spatial organization of the in vivo system and providing the possibility to visualize and analyze individual cells over time (Figure 3H) [78]. However, most cultures are short‐term, as lymphocytes typically egress from the slices within 1–2 days, although some show that tonsil slices can be informative for up to 4–7 days [34, 74]. Tonsil slice cultures revealed that FRCs can inhibit T cell activation in response to stimulation, a finding consistent with in vitro results [77]. However, the conditions that activated T cells in vitro were insufficient to activate them in tissue slices, highlighting clear discrepancies between the systems that may affect their translation in vivo [77].

## 5 DISCUSSION

While animal models have offered valuable insights into germinal center processes, they do not fully replicate human physiology and pathology. In contrast, human immune culture platforms, including in vitro and ex vivo models, present opportunities to deepen our understanding of the human adaptive immune response [39, 80].

Initial insights were gained via human 2D models, such as cell cocultures, whole‐blood assays, and microfluidic devices which offer simplicity, speed, and practicality for studying early T–B cell interaction in the GC process. However, these models are limited by their inability to replicate complex 3D interactions and microenvironments necessary for accurate in vivo representation [39, 43]. To overcome these limitations, 3D systems like organoids, organs‐on‐a‐chip, and ex vivo tissue cultures have been developed, as these systems better replicate tissue architecture [21, 34, 36, 39]. However, differences between these models are apparent and can impact their translational value.

Ex vivo whole tissue cultures offer the most accurate representation of the cellular composition, extracellular microenvironment, and spatial organization within the LN, but their short viability is a significant drawback [78]. Human tonsillar organoids, on the other hand, can be maintained for longer and important GC events, such as antigen‐specific antibody production, somatic hypermutation, class switch recombination, and the organization of dark and light zones can be replicated. Plasma cell differentiation in these models is uncertain [18, 46]. The absence of fluid flow in most immune organoids—except for bioreactor‐based models with perfusion systems—may limit their ability to fully support these cellular processes. With the use of on‐chip systems, biochemical and biomechanical cues like fluid flow, ECM components, and shear stress, are integrated and enhance their translational relevance. However, few are specifically designed to replicate the GC reaction, focusing instead on drug delivery and lymph node responses [65, 66]. While microfluidic LN‐on‐chip models mimic many GC events, clear GC‐like structures have yet to be conclusively demonstrated.

It is important to note that while germinal centers are typically found in secondary lymphoid organs, they are not restricted to these tissues. For instance, tertiary lymphoid structures (TLS) can form in response to chronic inflammation, such as in cancer [81]. These intratumoral TLS can recruit and activate T‐ and B cells, initiating a germinal center‐like response. The presence of TLS is associated with improved patient survival and better responses to immune checkpoint blockade therapy [81, 82, 83]. Therefore, the models discussed here could also be applied to study T‐ and B‐cell interactions within TLS.

All in all, current models offer valuable insights into T‐ and B‐cell interactions and the GC response, but no existing model fully recapitulates all aspects of the GC response and its intricate microenvironment. Current in vitro platforms fall short in microenvironment complexity, particularly in replicating somatic hypermutation and plasma cell differentiation. Thus, further research is needed to enhance these models and improve their translational accuracy to in vivo human lymph nodes.

## 6 FUTURE OUTLOOK

Most LN models currently replicate only specific aspects of the organ rather than providing a complete representation [84]. Accurately modeling the immune microenvironment is crucial for simulating the GC reaction, particularly because LN stromal cells are essential for lymphocyte survival, migration, and maintaining LN structure and function [42, 43]. However, these stromal cells cannot be sourced from blood, and current engineered cultures do not capture all stromal cell subtypes, limiting their ability to fully replicate the LN microenvironment [43]. Future advancements may include integrating lymphatic vessels and vascular models to better replicate microcirculation and cell migration, which could significantly impact immune response [39, 43]. Additionally, the development of complex LN‐multiorgan chip systems could revolutionize immunological research by mimicking interactions between lymph nodes and various organs [38]. Between these advances and the optimization of current models, there remains significant potential for improvement in creating comprehensive human‐based models for studying the GC response which could enhance our understanding of the human immune system and accelerate the development of effective vaccines and immunotherapies.

## Author Contributions

The conceptualization of the review was primarily led by Theo van den Broek, who formulated the study's initial ideas and objectives. Elisa Fleischmann conducted the initial literature review, gathering data and formal analysis with support from Vera Middelkamp. Elisa Fleischmann took the lead in visualizing the data to aid in interpretation. Theo van den Broek acquired the funding for the project, oversaw project administration, and provided supervision throughout the research process. Elisa Fleischmann took the lead in drafting the original manuscript, with contributions from Elisa Fleischmann and Vera Middelkamp in reviewing and editing the document to ensure accuracy and clarity.

## Conflicts of Interest

The authors declare no conflicts of interest.

## Permission to reproduce material from other sources

All figures were created with BioRender.

### Peer Review

The peer review history for this article is available at https://publons.com/publon/10.1002/eji.202451460

## Data Availability

Data sharing is not applicable to this article as no datasets were generated or analyzed during the current study.
